# Surveying the spatial distribution of feral sorghum (*Sorghum bicolor* L.) and its sympatry with johnsongrass (*S*. *halepense*) in South Texas

**DOI:** 10.1371/journal.pone.0195511

**Published:** 2018-04-26

**Authors:** Sara Ohadi, Matthew Littlejohn, Mohsen Mesgaran, William Rooney, Muthukumar Bagavathiannan

**Affiliations:** 1 Department of Soil and Crop Sciences, Texas A&M University, College Station, Texas, United States of America; 2 Department of Plant Sciences, University of California, Davis, United States of America; Instituto Agricultura Sostenible, SPAIN

## Abstract

Sorghum (*Sorghum bicolor*) is an important grain and forage crop grown across the US. In some areas, sorghum can become feral along roadsides and other ruderal areas, as a result of seed spill during harvest or transport. In some of these situations, feral sorghum grows in or near established johnsongrass (*S*. *halepense*) populations. Johnsongrass, a wild relative of sorghum and an incredibly noxious weed, is capable of hybridizing with cultivated sorghum. Because commercial hybrid sorghum cultivars are produced with cytoplasmic male sterility, progeny of the hybrid crop which compose the founder feral populations also segregate for male sterility. Consequently, male sterility in feral sorghum may increase the risk of outcrossing with johnsongrass. Using field surveys and spatial modelling, the present study aimed at documenting the occurrence of feral sorghum and understanding the anthropogenic and environmental factors that influence its distribution. Further, this research documented the sympatry of feral sorghum and johnsongrass in the roadside habitat. A total of 2077 sites were visited during a systematic field survey conducted in fall 2014 in South Texas. Feral sorghum and johnsongrass were found in 360 and 939 sites, while the species co-existed at 48 sites (2.3% of all surveyed sites). The binary logistic analysis showed a significant association between the presence of feral sorghum and road type, road body-type, micro-topography of the sampling site, nearby land use, and the presence of johnsongrass, but no association with the distance to the nearest grain sorting facility. The probability of finding feral sorghum away from johnsongrass patches was generally higher than finding them co-occur in the same location. A probability map for spatial distribution of feral sorghum was developed using the nearby land use type and the regional habitat suitability for johnsongrass as two key predictors. Overall, results show that feral sorghum and johnsongrass co-occur at low frequencies in the roadside habitats of South Texas, but these low levels still present a significant opportunity for hybridization between the two species outside of cultivated fields.

## Introduction

Escape and establishment of crop species outside of agricultural systems, known as *crop ferality*, can be a concern if the escaped crop contains novel traits and establishes self-perpetuating populations within agricultural landscapes [1–3]. A feral population can be found by the dispersal of seeds from the agricultural fields to adjacent habitats such as the roadsides. Human-mediated dispersal, such as seed spill from farm machinery (during planting and harvest) and seed transport trucks or hitchhiking on a vehicle, is the main vector for crop seed dispersal out of agricultural fields [4–5]. Animals (e.g. birds and rodents) can also disperse seeds, but to a lesser extent than humans both in terms of the distance and number of propagule that they can disperse [6].

The persistence of feral populations, similar to any plant population, can be influenced by the spatial and temporal heterogeneity of the habitat [7]. In a heterogeneous landscape, certain sites could be unfavorable for the establishment of feral populations [7] and at these sites the population may only sustain through continuous immigration of propagule from the source sites [8]. Arrival of large number of propagules would increase the survival chance of the population [8]. While propagule dispersal is a critical first step for the establishment of feral crop populations in natural areas, certain habitat characteristics such as vegetation density and drainage potential can also influence the establishment of feral populations in these areas [9]. Additionally, seed dormancy and the ability to establish a seedbank would allow the feral populations to persist in natural habitats and enable recovery from stochastic fluctuations of the environment [10–11].

Sorghum is a major crop in terms of production, ranking fifth worldwide and in the US [12]. As a drought tolerant crop, sorghum can be grown in areas where the extremes of high temperatures and low soil moistures are unsuitable for the production of other row crops such as corn [13]. Sorghum also has high potentials for development as a bioenergy feedstock [14–15]. Similar to many other feral crops [5, 16], sorghum may have the ability to establish self-sustaining populations outside of cultivated fields. The occurrence of feral crops along roadsides might be attributed to seed dispersal and the peculiarity of this habitat such as increased water runoff and low plant community richness [17]; these characteristics may favor the initial establishment of feral sorghum in roadside habitats. The ferality potential in sorghum will be of concern because of sympatric presence of weedy relatives that can outcross with cultivated sorghum. In Southern US, the weedy relatives of *S*. *bicolor* include shattercane (*S*. *bicolor* ssp. *drummondii*) and johnsongrass (*S*. *halepense*) [18]. Of these, johnsongrass is known to be the most widely distributed and frequently found relative of sorghum in South Texas (personal observations).

Johnsongrass is one of the most troublesome weeds in the world, capable of spreading by both underground rhizomes and seeds [19]. Johnsongrass can cause severe yield losses in sorghum and many other crops [20]. Both sorghum and johnsongrass are interfertile and can be hybridized under controlled conditions [21–24]. Gene flow from sorghum to johnsongrass has also been observed in natural conditions [25– 26]. For sorghum cultivars bred or engineered with adaptive or herbicide resistance traits, ferality can be a concern as it can facilitate the establishment of these traits in the broader environment, causing ecological and/or agronomic issues. The co-occurrence of feral sorghum and johnsongrass in the proximity would increase the chances of cross-pollination between the two species. Pollen-mediated gene flow from johnsongrass to sorghum may enhance ferality in sorghum through de-domestication and the provision of adaptive alleles. The majority of commercial sorghum cultivars grown in the US are hybrids, and cytoplasmic male sterility is used to produce hybrid seeds [27]. Male sterility is a recessive trait and male fertile F_1_ hybrids are actually in a heterozygous condition for alleles conditioning fertility restoration. Consequently, segregation for male sterility would be expected in progeny (ie, grain) from the hybrid. In fact, approximately 25% of the feral sorghum plants established through seed dispersal (F_2_ seed harvested in hybrid sorghum fields) will be male sterile. In these circumstances, the co-occurrence of feral sorghum and johnsongrass presents an increased chance for outcrossing.

Texas is the second largest sorghum producer in the US, closely following Kansas [28]. In South Texas, the majority of sorghum production is concentrated in the Rio Grande Valley, Coastal Bend and Upper Gulf Coast regions [28]. Sorghum grown in this region is frequently transported to Mexico along highways and railroads through the Rio Grande Valley. Sorghum seed spill along the transportation routes could lead to the establishment of feral sorghum populations in the byways along these routes and in fact, sorghum is seen commonly along the major highways in South Texas. However, no systematic survey has been conducted in the region to document the occurrence of feral sorghum along roadsides and the extent of sympatry with johnsongrass. The objective of this study was to document the prevalence of feral sorghum and johnsongrass along roadside habitats in South Texas and underpin, using GIS and logistic regression models, its association with several anthropogenic and environmental factors.

## Materials and methods

### Survey location and data collection

A field survey was conducted along the roadsides of South Texas, from the Rio Grande Valley to the Upper Gulf Coast where grain sorghum cultivation is prevalent. The climate of the survey region is humid subtropical with mild winters and warm summers [29]. Sorghum is planted from mid-February in the Rio Grande Valley to early April in the Upper Gulf Coast regions, with harvest occurring in about four months after planting. Due to the long growing seasons in South Texas, sorghum seed that disperses after harvest will have a chance to germinate and attain reproductive maturity prior to a killing frost (if one occurs) during late fall season. In the Rio Grande Valley, frost occurs very rarely and a second sorghum crop can be planted in early August. The survey was conducted during late October- early November 2014 to allow the feral sorghum along the roadsides to establish and mature. However, the survey also included feral sorghum plants that recruited during spring.

The survey area was divided into three regions based on distinct environmental conditions: 1) Upper Gulf Coast, from west of Houston, TX (29.7604° N, 95.3698° W) to Victoria, TX (28.8169° N, 96.9933° W), 2) Coastal Bend, from Victoria, TX to Kingsville, TX (27.5150° N, 97.8656° W), and 3) Rio Grande Valley, from Kingsville, TX to Brownsville, TX (25.9303° N, 97.4844° W). Survey sites within each region were chosen using a semi-stratified survey methodology, as described by Bagavathiannan and Norsworthy [30]. One hundred survey sites were selected at random within each region and survey routes were optimized using the ITN Converter software (Ver. 1.97, Benichou Software) on a Google^®^ map layer. The ITN files were loaded to a GPS device to facilitate navigation to the pre-determined survey sites. In each site, the presence/absence of feral sorghum and johnsongrass was recorded. If feral sorghum was present, observations were carried out on the feral population size and site characteristics within a 25 m strip along the roadside site (Table 1). If feral sorghum was absent in a pre-determined survey site, the first population found along the route to the next pre-determined site was used for characterization. No specific permissions were required for the activities carried out in this project. Moreover, the authors confirm that the field studies did not involve endangered or protected species.

**Table 1:** Details collected during the roadside survey for feral sorghum*.

Road type	Road body-type	Micro-topography	Nearby land use	Vegetation cover	Feral sorghum density	Johnsongrass
**County road**	Dirt	Road shoulder	Corn	0 (no vegetation)	1 (<5 plants)	0 (absent)
**Highway**	Gravel	Field shoulder	Cotton	1(1–10%)	2 (6–25 plants)	1 (present)
**Local street**	Paved	Field edge	Fallow	2 (11–20%)	3 (26–50 plants)	
**Federal road**			Hay	3 (21–30%)	4 (51–100 plants)	
**Functional classification (FC) street**			Herbs	4 (31–40%)	5 (> 100 plants)	
**Third-party toll road**			Urban	5 (41–50%)		
			Pasture	6 (51–60%)		
			Rice	7 (61–70%)		
			Shrub land	8 (71–80%)		
			Sorghum	9 (81–90%)		
			Soybean	10 (91–100%)		
			Sunflower			
			Turf			
			Wetlands			
			Wheat			
			Woods			

*Vegetation cover and feral sorghum population density were recorded only at sites where feral sorghum was present, within a 25 m strip along the roadside. The vegetation cover was recorded on a scale of 0–10 while the feral sorghum density was recorded on scale of 1–5.

At each site where feral sorghum was present, observations were also carried out on the road body-type (dirt, gravel or paved), micro-topography of the site (whether present at road shoulder, field shoulder and/or field edge), vegetation cover of the habitat (on a scale of 0–10), and nearby land use type (Table 1). The micro-topography category ‘road shoulder’ represents the area immediately adjacent to the road margin towards the deepest point of the ditch, ‘field shoulder’ represents the area from the deepest point of the ditch to the field edge. The ‘field edge’ represents the edge of the cultivated field. The road and field shoulders typically have high vegetation cover and minimally disturbed, whereas the field edges are often (but not always) tilled and have relatively less vegetation. The co-occurrence of feral sorghum and johnsongrass was defined when both species were present within 50 meters of each other. To understand whether there is a relationship between the presence of feral sorghum and distance to grain sorting facilities, locations of such facilities were recorded during the survey. Data pertaining to the nearby land use type and the presence/absence of feral sorghum and johnsongrass were used for developing a projection model for species distribution, as described below.

### Data analysis

**Fig 1:**
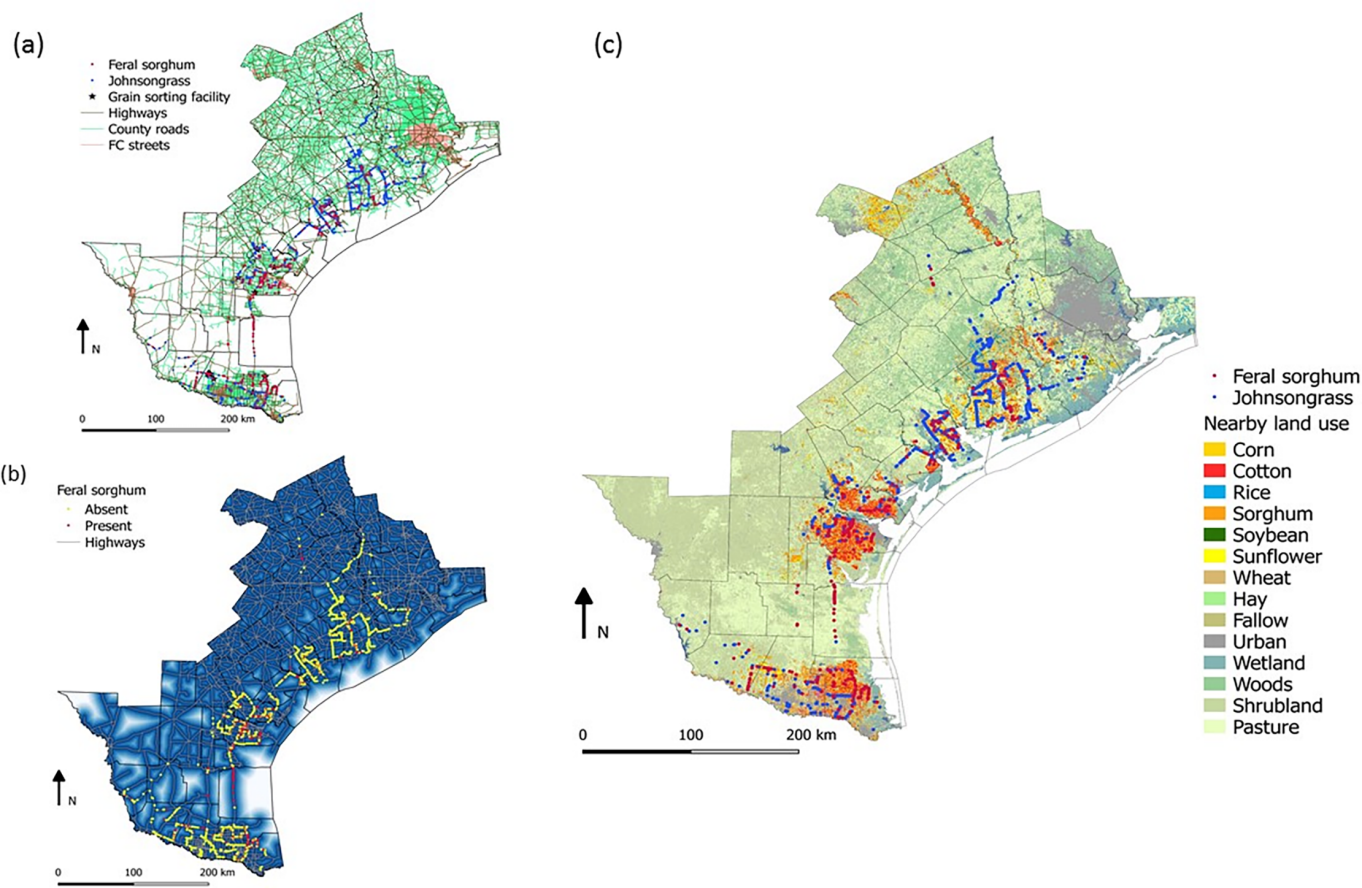
Maps of the different road types in association with the presence of feral sorghum and johnsongrass (a), the raster map showing the distance of each pixel from its nearest highway (b), and GPS waypoints of feral sorghum and johnsongrass relative to land cover data obtained from CropSpace (c). Abbreviation: FC street, Functional Classification street.

For each sampled site, the nearest road type was identified using Texas road maps (TxDOT Roadways) obtained from Texas Natural Resources Information System (TNRIS) online database (https://tnris.org/data-catalog/entry/txdot-roadways). The road type classifications were county roads, highways, local streets, federal roads, functional classification (FC) streets and third-party toll roads. However, the recorded feral sorghum populations were predominantly found in county roads, highways and FC streets and not the other road type categories. As shown in Fig 1, only a sub-region of South Texas, representing the latitudinal and longitudinal limits of anticipated feral sorghum distribution, was included in the analysis. To determine the nearest road type to a given sample site, we first converted the road vector lines to a raster format using the Qgis software (version 2.18). Prior to rasterization, the character strings were converted to numerical values; for example, a highway road type was assigned a numeric value of 1while a county road was given a value of 2, and so forth. For each individual road type, a proximity map was produced which gives the distance of each grid cell on the map from the given road type (Fig 1A and 1B). The georeferenced sampled sites were then overlaid on these road proximity maps and the raster value of each individual proximity map was appended to these points sequentially. The nearest road type to a given sample site was determined as the road with the smallest proximity (distance) value.

To determine the nearby land use for the sampled points, we used the very high resolution (30 m) CropScape database [31]. We drew a buffering circle with a radius of 90 m around each sampling point and then converted the resultant circle polygons to a raster format using the values of CropScape raster layers as the required data field for the conversion. Similar to road type data, nearby land use data are presented using numerical values; for example, a sorghum crop is identified by the value ‘4’ (a total of 254 numerical values were identified for land cover in the CropScape database). Using the Zonal Statistical feature of Qgis, the mode was calculated for each circle: mode represents the land cover with the highest frequency for the circle and thus dominant crop (or other land uses) adjacent to the sampled sites. The distance of the sampling site to the nearest grain handling facility was calculated using the Matrix Distance feature of Qgis.

Using a binary logistic model, we modelled the probability of the presence of feral sorghum as a function of the road type (highways, county roads and functional classification streets), road body-type (dirt, gravel or paved), micro-topography of the surveyed site (road shoulder, field shoulder, field edge), the nearby land use (corn, cotton, fallow, hay, herbs, urban, pasture, rice, shrubland, sorghum, soybean, sunflower, turf, wetlands, wheat and woods), presence/absence of johnsongrass and distance to grain sorting facilities. The above model was fitted using the PROC LOGISTIC procedure of SAS (version 9.4). The ‘odds ratio’ feature of PROC LOGISTIC was used to calculate the odds ratio and test for significance of the differences between the levels of predictors. Prior to analysis, all data were reordered to reduce the number of categories and avoid the effect of ‘quasi-complete separation of data points’. For example, 40 categories were initially recorded for nearby land use but were then reclassified to 16 groups. At 360 sites (i.e. sites that feral sorghum was present), the density of the feral sorghum population was recorded on scale of 1 to 5 (see Table 1). The effects of the road type, road body-type, micro-topography of the site, the nearby land use, the vegetation cover and presence/absence of johnsongrass on the scores of feral population density was investigated using PROC GLM of SAS (version 9.4) and means were separated using the least significant differences (LSD) test.

### Spatial model

A spatial model was developed to map the potential distribution of feral sorghum in the south Texas. Our initial analysis showed that the probability of finding feral sorghum at a given site is strongly associated with the nearby land use type and the occurrence of johnsongrass. For this purpose, we used a johnsongrass habitat suitability map produced based on broader climate variables (cordially provided by Dr. Daniel Atwater). For each sample site, we extracted the suitability values from this map and the nearby land use from CropScape [31] as described above. A binary logistic model was then fitted using the feral sorghum presence/absence data as the dependent (response) variable while land use and habitat suitability (for johnsongrass) as the independent (predictors) variables. The parameter estimates from this model were inserted into the Raster Calculator of Qgis to obtain the probability values of feral sorghum occurrence across South Texas. The output of the binary logistic model are given in logit unit; to transform these data to original unit (i.e. probability values ranging from 0 to 1), we used $P=\frac{exp(L)}{1+exp(L)}$, where *L* is the predicted value in logit and *P* is its respective back-transformation value in probability unit.

## Results and discussion

### Occurrence of feral sorghum

A total of 2,077 sites were visited for the presence of feral sorghum and johnsongrass in our survey. Feral sorghum was found in 17% (360) of the sites, whereas johnsongrass was more abundant and found in 45% (939) of the sites visited. To our knowledge, this is the first documented account of the occurrence of sorghum as feral populations outside of cultivated fields. Results from the logistic model showed a significant association between the presence of feral sorghum with the road type and its body-type, micro-topography of the site, nearby land use and the presence/absence of johnsongrass, but showed no relationship with distance to the nearest grain sorting facility (Table 2).

**Table 2:** The result of binary logistic analysis for testing the significance of the explanatory variables on the probability of feral sorghum occurrence in South Texas.

Factors	Df	Chi-Square	*P*
Road body type	2	4.946	< .0001
Road type	2	7.79	0.0204
Micro-topography of the site	2	162.25	< .0001
Nearby land use	15	56.68	< .0001
Presence of johnsongrass	1	69.86	< .0001
Distance to grain sorting facility	1	1.05	0.3048

**Fig 2:**
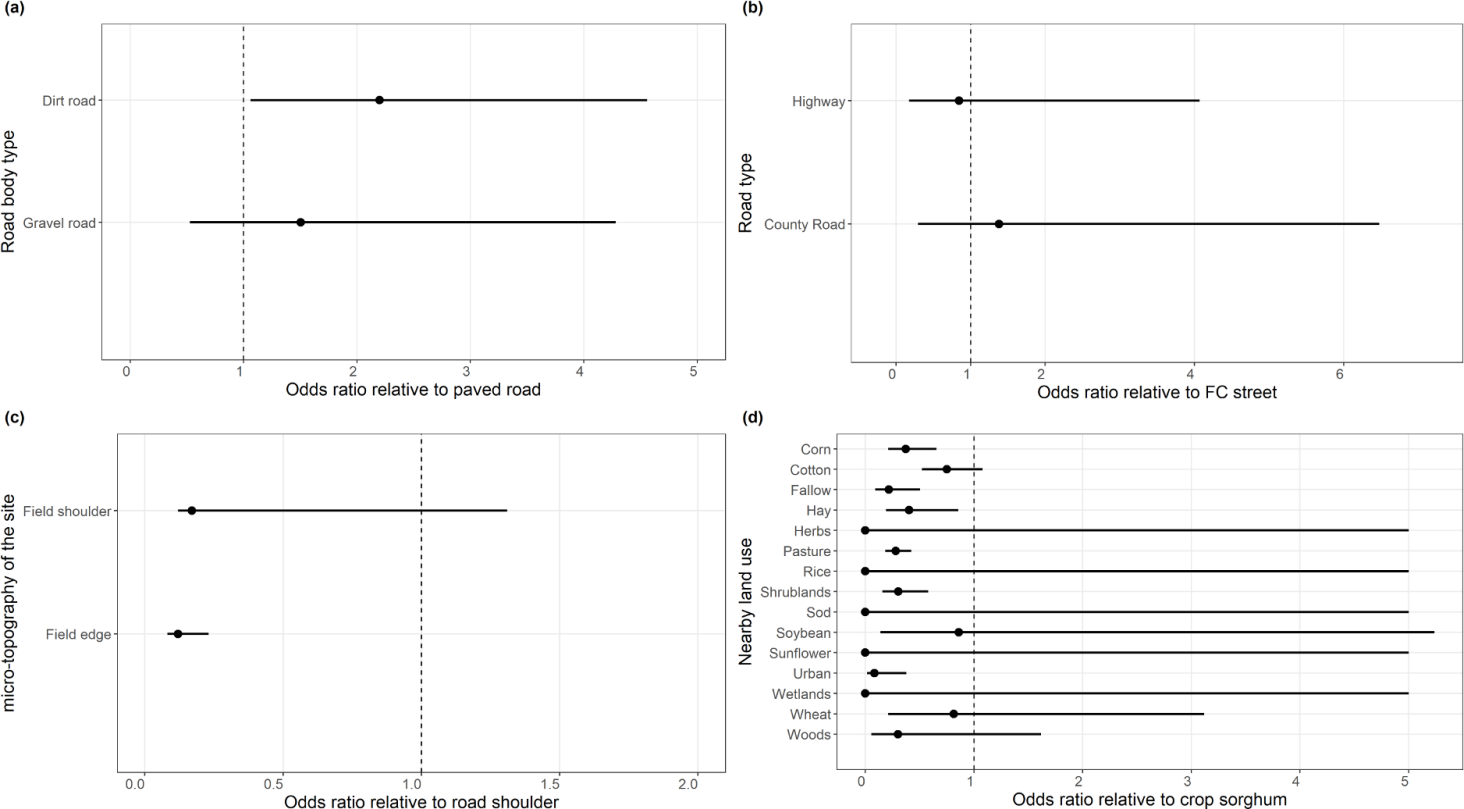
Odds ratio value (black dot) for the effects of road body-type (a), road type (b), micro-topography of the surveyed site (c) and nearby land use (d) on the presence of feral sorghum. The solid black line indicates the 95% confidence intervals for the odds ratio.

The odds ratio analysis showed that the likelihood of occurrence of feral sorghum along a gravel or dirt road is 1.5 or 2.4 times greater than that of a paved road, respectively (Fig 2A). Dirt and gravel roads are prevalent in rural areas surrounding farmlands and high likelihood for the presence of feral sorghum populations along these road types suggests that movement of farm equipment and production activities greatly contribute to sorghum seed dispersal into roadside habitats. Further, washboarding, corrugation and any potholes on the surface of unpaved roads can increase vehicle bouncing and thus increase the chances of seed spill from seed transport trucks and farm equipment. Contrary to our findings, the higher frequency of feral oilseed rape along the paved compared to the gravel and dirt roads in France was attributed to the higher traffic intensity with commodity transport on paved roads [32]. Although the Chi-square test suggested significant differences between the road types (Table 2), the odds ratio analysis failed to detect such differences (Fig 2B). This may suggest that all road types are equally likely to accommodate feral sorghum, following a seed immigration event. Although previous studies have found a strong relationship between the presence of feral crops and road type [9, 32–33], this was not the case for feral sorghum in South Texas. One notable exception in the present survey was that feral sorghum populations were common along U.S. Highway 77 between Kingsville and Raymondville, TX where sorghum and crop production in general is very sparse. The high intensity of grain transport via truck movement (personal observations) is likely contributing to sorghum seed dispersal along this highway.

Feral sorghum was more likely to be present at the road shoulders (53% of the sites) as shown by the odds ratio values (Fig 2C), but they were also present in the field shoulders (25% of the sites) and at field edges (23% of the sites). However, as the road and field shoulders are only separated by few meters in many cases, feral sorghum found along the road verges could also be sourced by sorghum production activities in the adjacent fields. This postulation is supported by a lack of significant difference between the odds of feral sorghum presence at field shoulders relative to that of the road shoulders, as shown by the odds ratio estimates (Fig 2C). However, a logical question could be why feral sorghum is less abundant at field shoulders or field edges if the adjacent fields could contribute to propagule immigration. Field edges are typically disturbed (e.g. plowed, sprayed), disrupting the establishment and persistence of feral sorghum in these sites. Nevertheless, the microhabitats at the roadside could provide more moisture for the establishment of plants compared to field edges [9].

The nearby land use had large effects on the presence of feral sorghum (Table 2). Results showed that the odds for the occurrence of feral sorghum in sites adjacent to sorghum cultivation was larger than that of all other land uses; the likelihood of finding feral sorghum in a location closer to a sorghum field was almost twice as high as a location contiguous to corn, hay, pasture, shrubland, urban or fallow lands (Fig 2D). Sorghum is one of the major crops grown in South Texas and results suggest that sorghum cultivation and seed transport activities in the region contribute to seed immigration and establishment of feral sorghum on roadside habitats. Further, the sorghum seed dispersed following harvest might germinate instantly due to the lack of seed dormancy and the warm environmental conditions in South Texas may allow feral sorghum to produce viable seed prior to killing frost and establish self-perpetuating populations. There is also a possibility for spring establishment of feral sorghum from the seeds entered into the soil post-harvest should they be able to survive during the fall and winter. Data on seed survival rate of sorghum coupled with early spring monitoring are needed to address this question.

### Population size of feral sorghum

**Fig 3:**
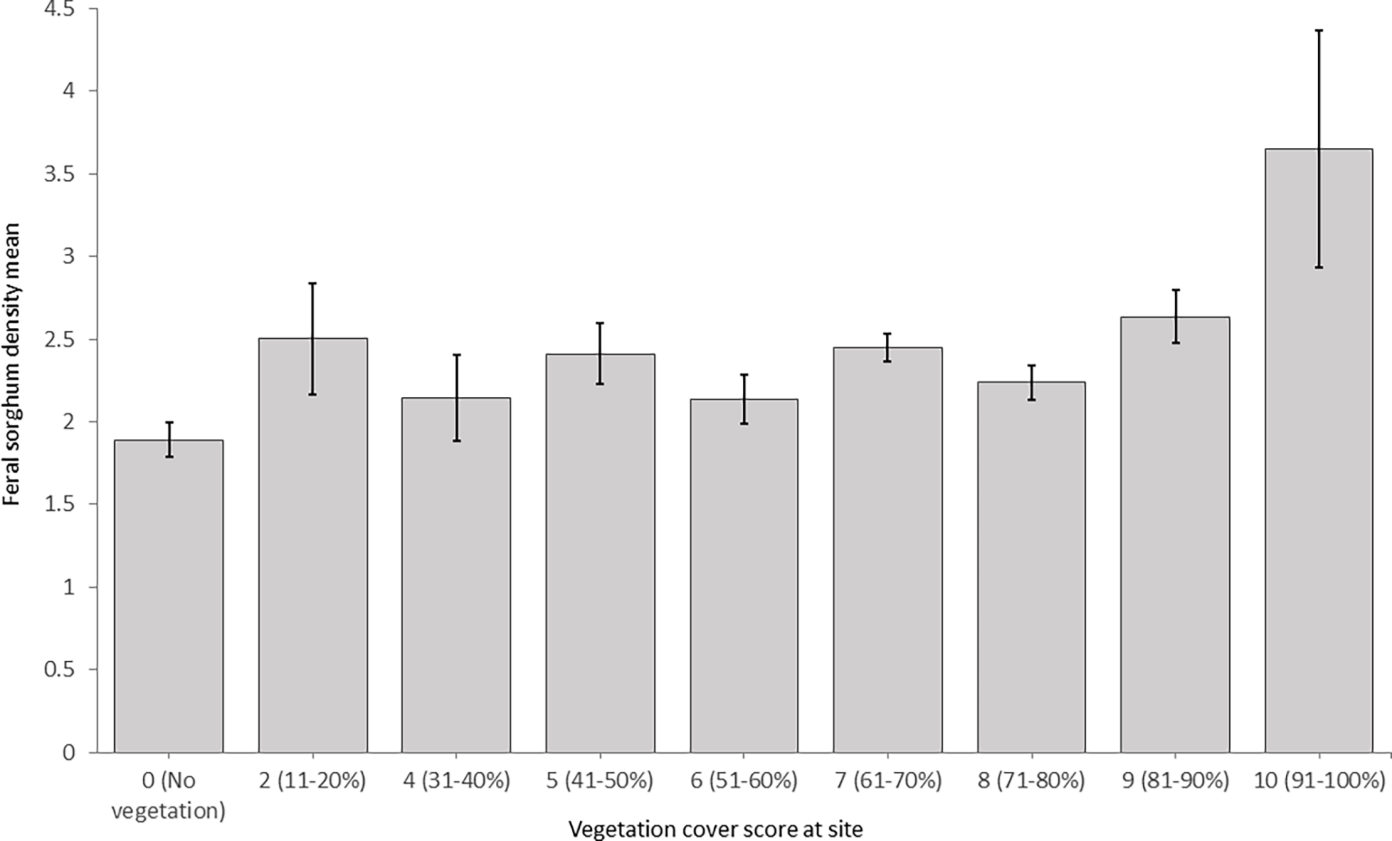
Impact of roadside vegetative cover on the occurrence of feral sorghum populations.

The population size of the feral sorghum at each site was scored (Table 1) based on visual estimations. The results from the analysis of variance showed no significant relationship between all the measured factors and the population size of the feral sorghum at sampled sites expect for the vegetation cover (Table 3). The largest feral sorghum population sizes were associated with the highest vegetation cover (>90% ground cover) (Fig 3), a finding that is unexpected given that vegetation with higher canopy cover should be more resistant to invasion than those with low canopy cover [34]. One possible explanation is that the roadsides (especially highways) are regularly treated with herbicides by the Department of Transportation and in some cases by county weed control specialists for controlling tall vegetation such as johnsongrass. It is likely that herbicides might have been recently applied at sites with low vegetation cover, thus reducing the chance of observing feral sorghum individuals.

**Table 3:** Result of the analysis of variance testing the significance of the explanatory variables on feral sorghum population size in South Texas.

Factors	Df	Mean square	*P*
Road type	2	0.885	0.323
Road body-type	2	1.062	0.258
Micro-topography of the site	2	1.582	0.133
Nearby land use	8	1.205	0.141
Vegetation cover at site	8	2.497	0.0017
Distance to grain sorting facility	1	0.291	0.541
Presence of johnsongrass	1	0.488	0.429

### Co-occurrence of feral sorghum and johnsongrass

**Fig 4:**
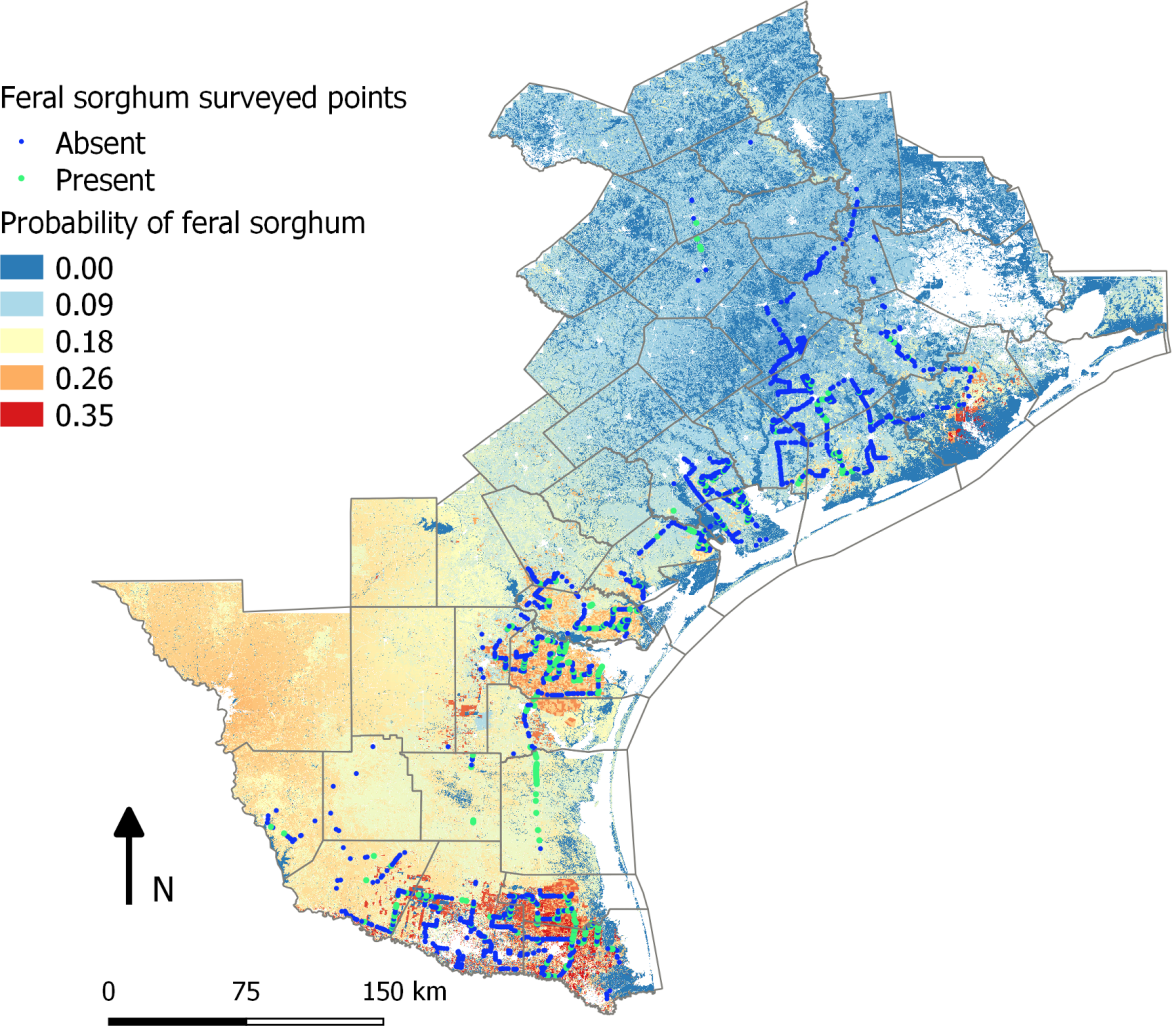
Example of a feral sorghum-johnsongrass complex site along a roadside near Corpus Christi, TX.

The co-occurrence of feral sorghum and johnsongrass was rare and both species were found together only in 48 of the 2,077 survey sites visited (i.e. 2% of the sampled sites). Fig 4 shows co-occurrence of feral sorghum and johnsongrass in a roadside site near Corpus Christi, TX. Results from the logistic model showed a negative relationship between the occurrence of johnsongrass and feral sorghum (Table 2). The likelihood of detecting feral sorghum at locations without johnsongrass was 4.3 (confidence intervals: 3.059–6.068) times greater than the locations where johnsongrass was present. Three possible scenarios might explain this finding: (1) the presence of johnsongrass in the site may have a negative influence on the germination and establishment of feral sorghum (such as the production of allelochemicals); however, the data collected in this study was not sufficient to establish any causal relationship or there is no anecdotal evidence to support such a scenario in production fields, (2) the dispersal of sorghum seed on roadsides as a function of intensive sorghum cultivation (Fig 1C) and seed transport occurs primarily in the much Southern parts of Texas from Victoria towards Brownsville, an environmental gradient increasingly less suited for johnsongrass (evident in our surveys (Fig 1C) as well as in the habitat suitability map for johnsongrass); thus, the co-occurrence of both species was perhaps naturally limited, and/or (3) roadside herbicide applications that target johnsongrass may eliminate any feral sorghum plants present within these sites, while johnsongrass could regrow from rhizomes. It is very likely that the second and third scenarios have substantial influence on the co-occurrence of these two species. A follow-up observation conducted in summer 2017 has revealed supporting evidence for the third scenario in that several of the feral sorghum-johnsongrass complex sites we identified in the 2014 survey were severely impacted by roadside herbicide applications that typically target johnsongrass. In these sites, several johnsongrass plants survived, but almost all feral sorghum plants were eliminated.

Given that feral sorghum and johnsongrass can hybridize, the co-occurrence of these two species may facilitate the persistence of feral sorghum through gene flow and introgression of adaptive traits from johnsongrass. In addition, cultivation of diverse sorghum lines, including sudangrass and sorghum-sudangrass hybrids, in the vicinity may enrich the diversity within the feral sorghum populations and thereby increase the adaptive ability of feral sorghum. Such an outcome has been reported for feral populations of oilseed rape (*Brassica napus*) and alfalfa (*Medicago sativa*) [35–36]. Since this is the first record of the presence of feral sorghum in nature, no information is available on the diversity, population genetic structure and long-term persistence of feral sorghum populations.

### Potential distribution of feral sorghum

**Fig 5:**
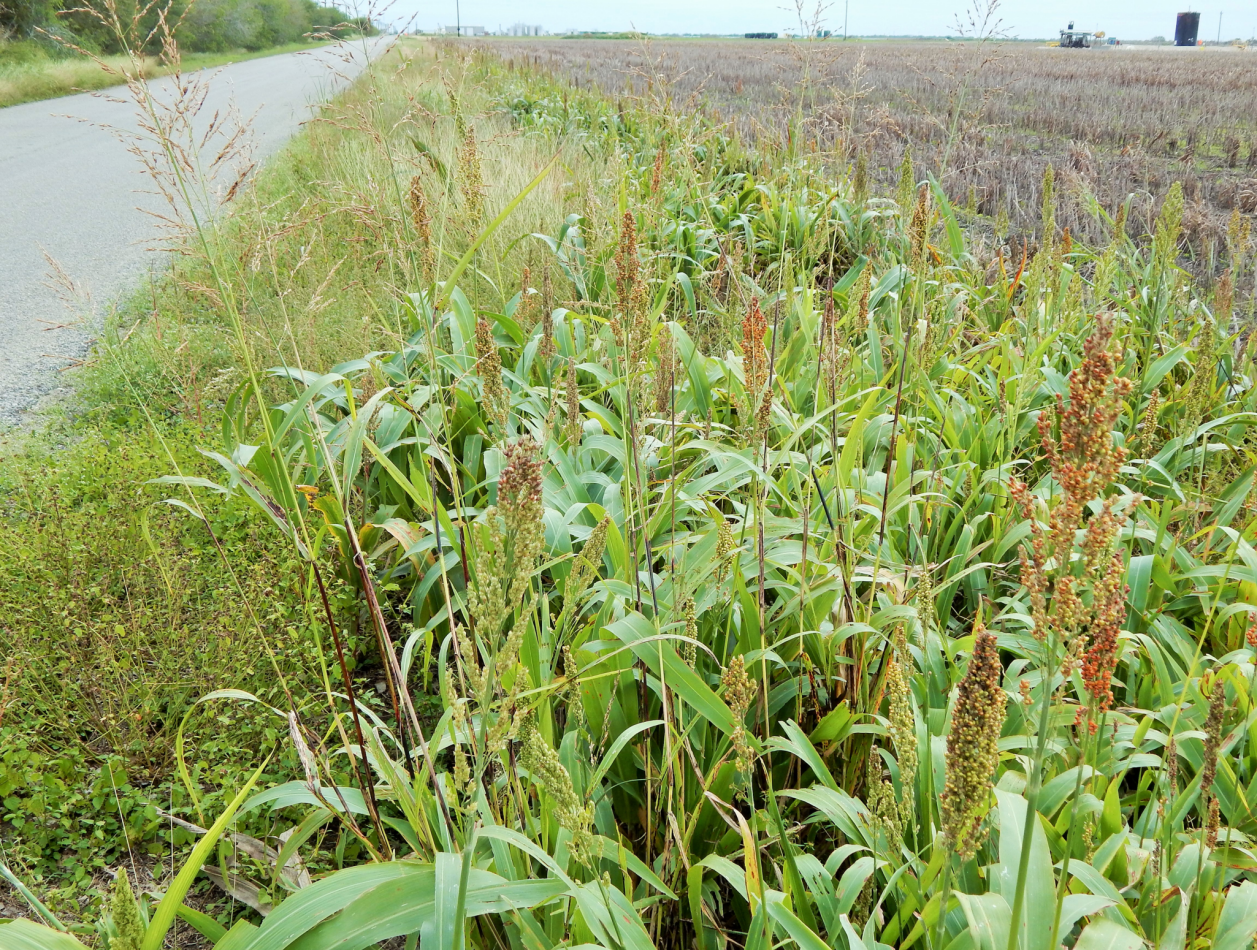
Map representing the probability of feral sorghum occurrence based on the nearby land use and johnsongrass habitat suitability.

Although most of the feral sorghum populations were observed along the roadsides, they may have the potential for spread to their contagious natural and unmanaged areas. To investigate the potential for broader distribution of feral sorghum in South Texas, we calibrated a model using nearby land use type and regional habitat suitability for johnsongrass as reliable predictors. These two variables were chosen because they were statistically significant and the georeferenced data for the entire region was available. The combination of these two variables effectively predicted the distribution of feral sorghum, with an increasing trend in abundance from the Upper Gulf Coast towards the Rio Grande Valley, which corresponded to an increasing intensity of sorghum cultivation (Fig 1C) and seed transport activities in the landscape. Further, in the more Southern areas of Texas, sorghum seeds germinating after the harvest season will have a high chance to produce mature seed prior to killing frost, if any. The projected map for feral sorghum distribution is shown in Fig 5, which corroborates with the overall trend observed in the survey. Conversely, the distribution of johnsongrass showed an opposite trend, with more abundance in the Upper Gulf Coast region than in the Rio Grande Valley (Fig 1C), attributable to its habitat suitability as evident in the habitat suitability map (not shown).

## Conclusions

Roadsides are known to be the main corridors for the escape of crops away from the agricultural fields. The current survey showed that roadsides and field margins are the initial niches for feral sorghum to establish outside of cultivated fields. We found that the occurrence of feral sorghum in South Texas is highly associated with sorghum cultivation in the nearby area, providing propagules for the establishment of feral populations in field edges and roadsides during planting and grain transport operations. We did not find any relationship between the frequency of feral sorghum and road characteristics (i.e. road type and body type). Although johnsongrass can be found commonly along the roadsides in South Texas, the co-occurrence of feral sorghum and johnsongrass was infrequent. Yet, there are significant opportunities for outcrossing to occur between the two species outside of cultivated fields. More research is necessary to understand the frequency of outcrossing between the two species and fitness of the progenies. Experiments are on the way to characterize, using phenotypic and molecular markers, the progeny of seed harvested from feral sorghum plants during this survey in sites where both species co-existed. Further, field surveys and monitoring are being carried out to confirm and characterize potential hybrid progenies in nature in these feral sorghum-johnsongrass complex sites.
