# Correction: Surveying the spatial distribution of feral sorghum (*Sorghum bicolor* L.) and its sympatry with johnsongrass (*S*. *halepense*) in South Texas

**DOI:** 10.1371/journal.pone.0200984

**Published:** 2018-07-16

**Authors:** Sara Ohadi, Matthew Littlejohn, Mohsen Mesgaran, William Rooney, Muthukumar Bagavathiannan

In the Author Contributions section, Mohsen Mesgaran (MM) should be listed as one of the persons who contributed to Writing–review & editing.

The legends for Figs 4 and 5 are incorrectly switched. Please see the correct Figs 4 and 5 here.

**Fig 4:**
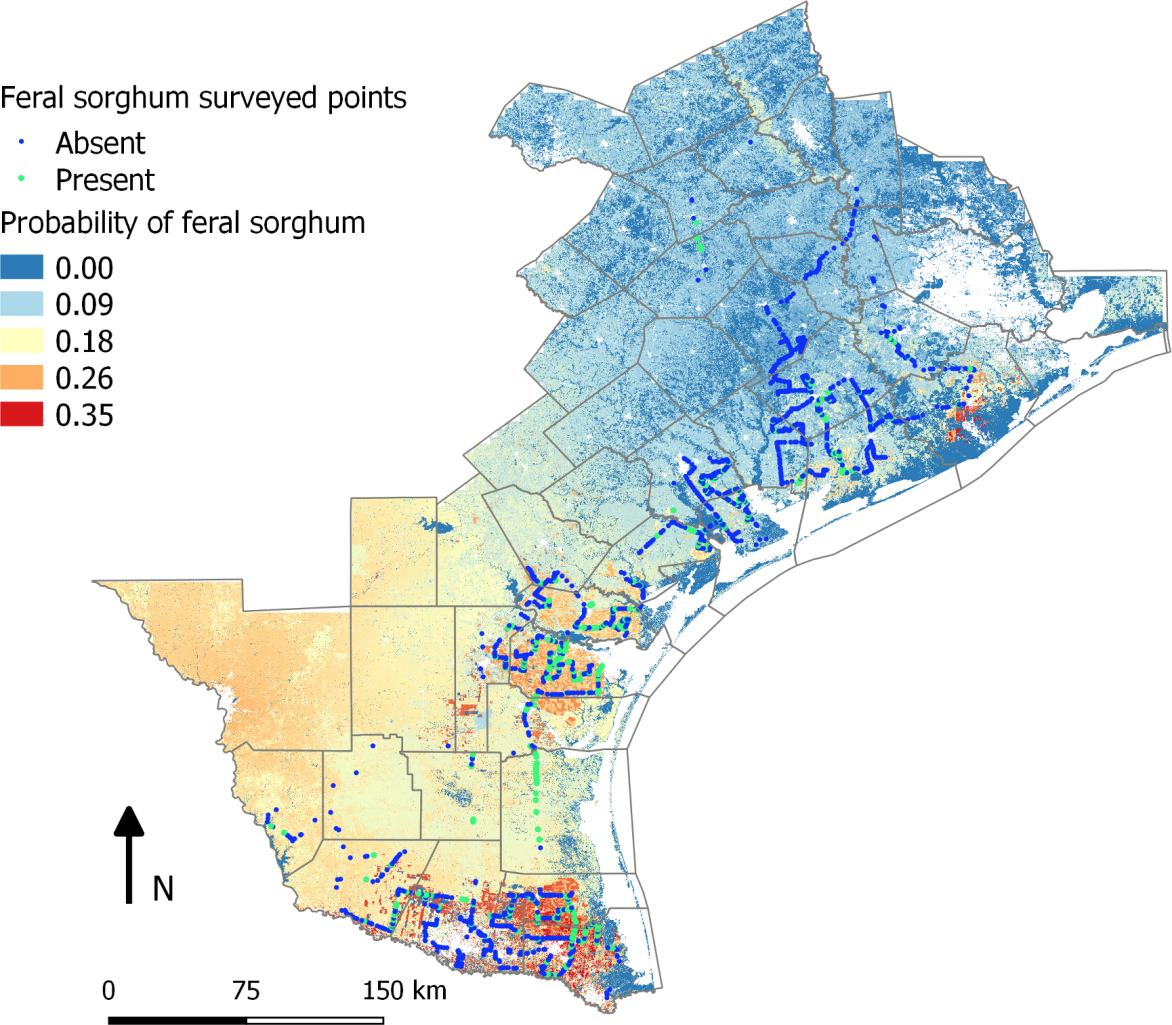
Map representing the probability of feral sorghum occurrence based on the nearby land use and johnsongrass habitat suitability.

**Fig 5:**
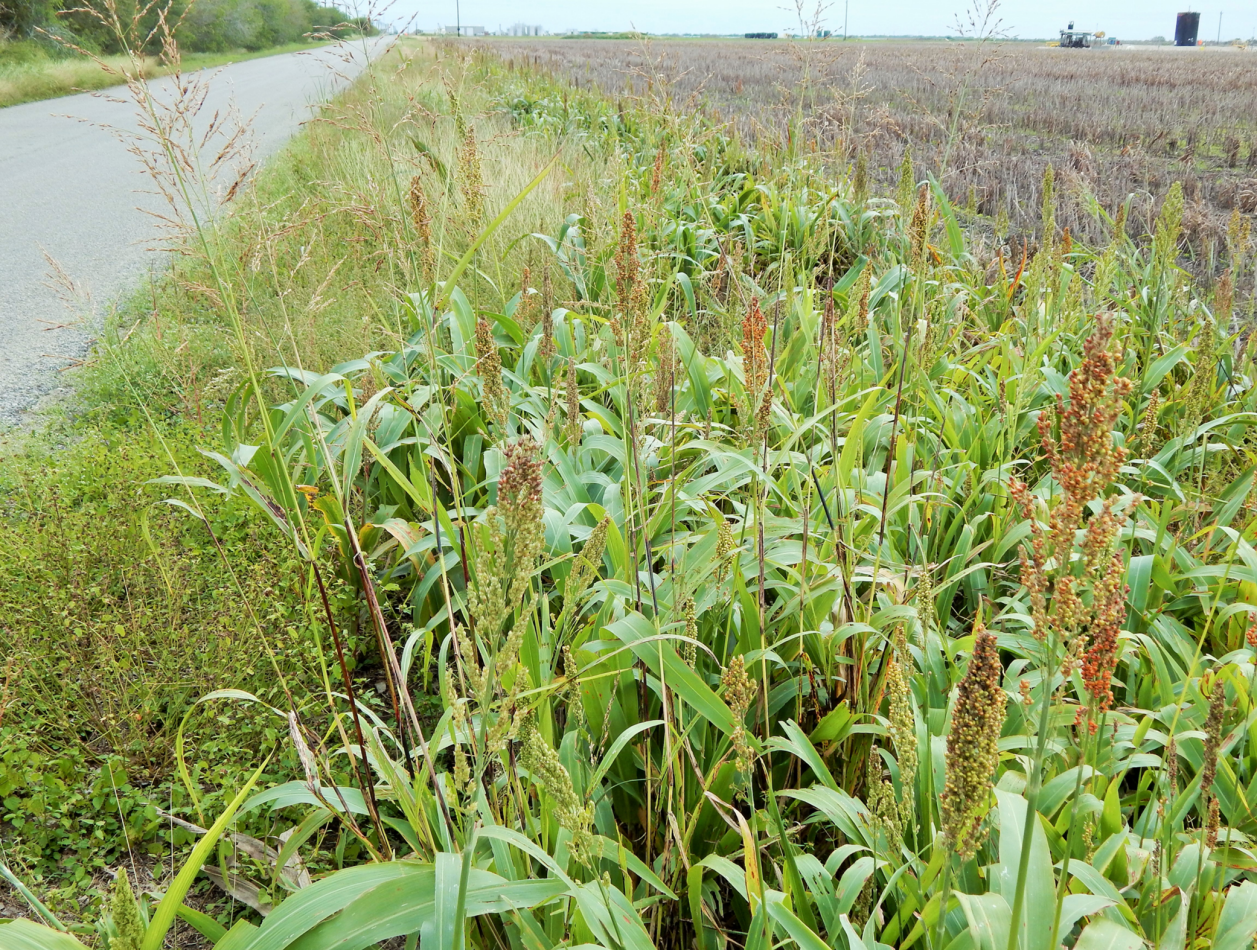
Example of a feral sorghum-johnsongrass complex site along a roadside near Corpus Christi, TX.
